# Collective departures and leadership in zebrafish

**DOI:** 10.1371/journal.pone.0216798

**Published:** 2019-05-23

**Authors:** Bertrand Collignon, Axel Séguret, Yohann Chemtob, Leo Cazenille, José Halloy

**Affiliations:** Laboratoire Interdisciplinaire des Énergies de Demain, UMR 8236, Université Paris Diderot, Sorbonne Paris Cité, 75013, Paris, France; University of California Santa Barbara, UNITED STATES

## Abstract

In social animals, morphological and behavioural traits may give to some individuals a stronger influence on the collective decisions, even in groups assumed to be leaderless such as fish shoals. Here, we studied and characterized the leadership in collective movements of shoals of zebrafish *Danio rerio* by observing groups of 2, 3, 5, 7 and 10 zebrafish swimming in a two resting sites arena during one hour. We quantified the number of collective departures initiated by each fish and the number of attempts that they made. To do so, we developed an automated pipeline that analysed the individual trajectories generated by the tracking software. For all shoal sizes, the leadership was distributed among several individuals. However, it was equally shared among all the fish in some shoals while other groups showed a more asymmetrical sharing of the initiation of collective departures. To quantify this distribution, we computed the entropy associated with the time series of the identity of all initiators for each experiment and confirmed the presence of a continuum between a homogeneous and a heterogeneous distribution of the leadership. While some fish led more departures than others, an individual analysis showed that all fish had actually the same success rate to lead the shoal out of a resting site after an attempt. Thus, some individuals monopolized the leadership by attempting more often than others to exit a resting site. Finally, we highlight that the intra-group ranking of a fish for the initiative is correlated to its intra-group ranking for the average speed with mobile individuals more prone to lead the shoal. These results demonstrate that the collective behaviour of a shoal can be mainly driven by a subset of individuals even in the absence of higher influence of a fish on its congeneers.

## Introduction

Collective movements in animals often require that the group members make decision on the move. In this process, an individual generally initiates the movement of the group towards a new direction or out of a resting site. The identity and motivation of this initiator vary widely according to the social organisation of the considered species [1, 2]. The initiation of movement may be undertaken by a unique or a subset of individuals resulting in a consistent leadership over time. These individuals can be older [3], of a specific sex [4] or dominant in the group [3, 5]. In societies that do not identify a specific individual as the group leader, initiations may be performed by any member of the group without consistency over time. In this context, fish are a traditional example of animal that form leaderless shoals or schools and rely on self-organized processes for information transfer and collective decision-making [6–9]. Indeed, a distributed mechanism in which any individual can potentially initiate a collective movement seems particularly suited for large schools of fish that lack global communication systems and share similar interests and costs [10, 11]. For example, the first fish to spot a predator coming potentially from any direction can start an escaping manoeuvre that will be propagated from neighbour to neighbour in the whole school.

In this case, the emergence of a leader is favored by external factors (e.g. the direction of the predator’s attack and it’s perception). However, internal factors can also cause an individual to act as a leader. Indeed, these individuals may be temporarily more motivated due to their physiological state [12–15], level of information [7, 16, 17] or position in the group [18, 19]. In fish shoals, collective movements are mainly driven by the individuals located at the front of the shoal [18]. Several motivations might prompt a fish to occupy these leading positions. Starved fish that have temporary higher nutritional needs are observed at the front positions of the shoal [20] associated with a higher rate of prey capture and food intake [21, 22]. In this case the preference for leading positions dissipates once the fish are fed [20]. Similarly, individuals that know the location of a potential food source can lead a group of naive fish towards foraging patches either by initiating departures [23] or favoring a particular swimming direction [7, 24]. The success of this steering has been shown to be related to the size of the guiding individuals in golden shiners, larger individuals being more often followed than smaller ones [25]. In this case, the propensity of some fish to take the lead is related to an information that can be gained by other fish or that can become outdated, resulting in an ephemeral leadership by some group members. Finally, the initiation of collective departures has been related to the personality of the fish (mainly *bold* versus *shy*) by several studies. Indeed, while front positions are linked with higher food intake, they are also more exposed to attacks by ambushed predators [26]. Faced with this trade-off, bolder individuals are more prone to exit a shelter and search for food than shyer fish that will mostly follow them rather than initiate a departure [27]. This asymmetry can be reinforced by the social composition of the group with shy individuals enhancing leadership b y bold ones [28]. In addition, bolder individuals show a lower behavioural plasticity than shyer ones, even when rewarded after following a partner rather than taking the lead [29, 30]. Thus, although each individual can initiate a collective movement, some characteristics may enhance the probability of some fish to take the leadership more often than others.

While the literature provides evidences for morphological and behavioural traits that lead some fish to become initiator more than others, the impact of this heterogeneous distribution of initiative on the collective dynamics of the group remains unclear. Indeed, most of the works rely on a preliminary binary classification of the individuals (e.g. bold or shy) that are then observed only in pairs with both fish being physically separated in two adjoined tanks or on the observation of groups during a short period of time, often due to tracking limitations preventing a reliable identification of the fish. Therefore, the relation between the individual characteristics of the fish and the distribution of the leadership is still clouded by the lack of repeated observations of collective departures in freely swimming shoals. However, the recent development of tracking techniques based on the individual recognition of specific patterns associated with each fish [31] allows us to overcome these limitations and to individually follow fish in larger groups and for longer time periods.

In this context, we studied the distribution of leadership during collective movement in small shoals of zebrafish *Danio rerio* swimming in an environment composed by two connected spots. At the collective level, the shoaling behaviour of zebrafish is already observed in larvae and shoaling preferences appear at the juvenile stage [32]. Once adult, zebrafish periodically oscillate from loosely connected groups to dense aggregates [33] and regularly transit from unstructured shoals to polarised schools (and inversely). During the school phases, they show a larger inter-individual distances and swim at a higher speed [34]. Here, we observed shoals of 2, 3, 5, 7 and 10 zebrafish swimming for one hour in an experimental arena consisting in two rooms connected by a corridor. We expected the fish to show a succession of mobile and static phases with frequent transitions from one spot to the other one, as observed for other heterogeneous environments [35]. For each experiment, we measured the number of collective transitions from one site to the other as well as the identity of the leading fish of each departure. In addition, we also quantified the number of attempts to initiate a collective departure made by each fish. As fish are generally characterized as leaderless, we expect that the number of initiated departures is proportional to the number of attempts made (i.e. the fish have a similar success rate). However, as mentioned above, individualities may incline some fish to initiate a collective departure more often than other ones. In particular, we expect that the individuals with the highest mobility would be more likely to steer the shoal by attempting to leave a resting site more frequently than the other members of the shoal. Therefore, we also characterized the mobility of the fish by computing their average speed as well as the related intra-group ranking from the fastest fish to the slowest one and related these traits with their tendency to lead their shoal out of the resting sites.

## Materials and methods

### Ethic statement

The experiments reported in this study were approved by the Buffon Ethical Committee (registered to the French National Ethical Committee for Animal Experiments #40) after submission to the state ethical board for animal experiments.

### Animals and housing

Around 150 adult laboratory wild-type zebrafish (*Danio rerio* AB strain) were reared in housing facilities ZebTEC and fed two times a day (Special Diets Services SDS-400 Scientific Fish Food). The sex ratio was close to 1:1 with females and males randomly mixed in groups of 15 to 20 fish housed in 8l containers with continuous renewal of the water by the ZebTEC system. All zebrafish observed in this study were 6-12 months old at the time of the experiments. We kept the fish under laboratory conditions, 27℃, 500μS salinity with a 10:14 day:night light cycle. The water pH was maintained at 7 and the nitrite concentration (NO^2-^) was below 0.3 mg/l.

### Experimental setup

**Fig 1:**
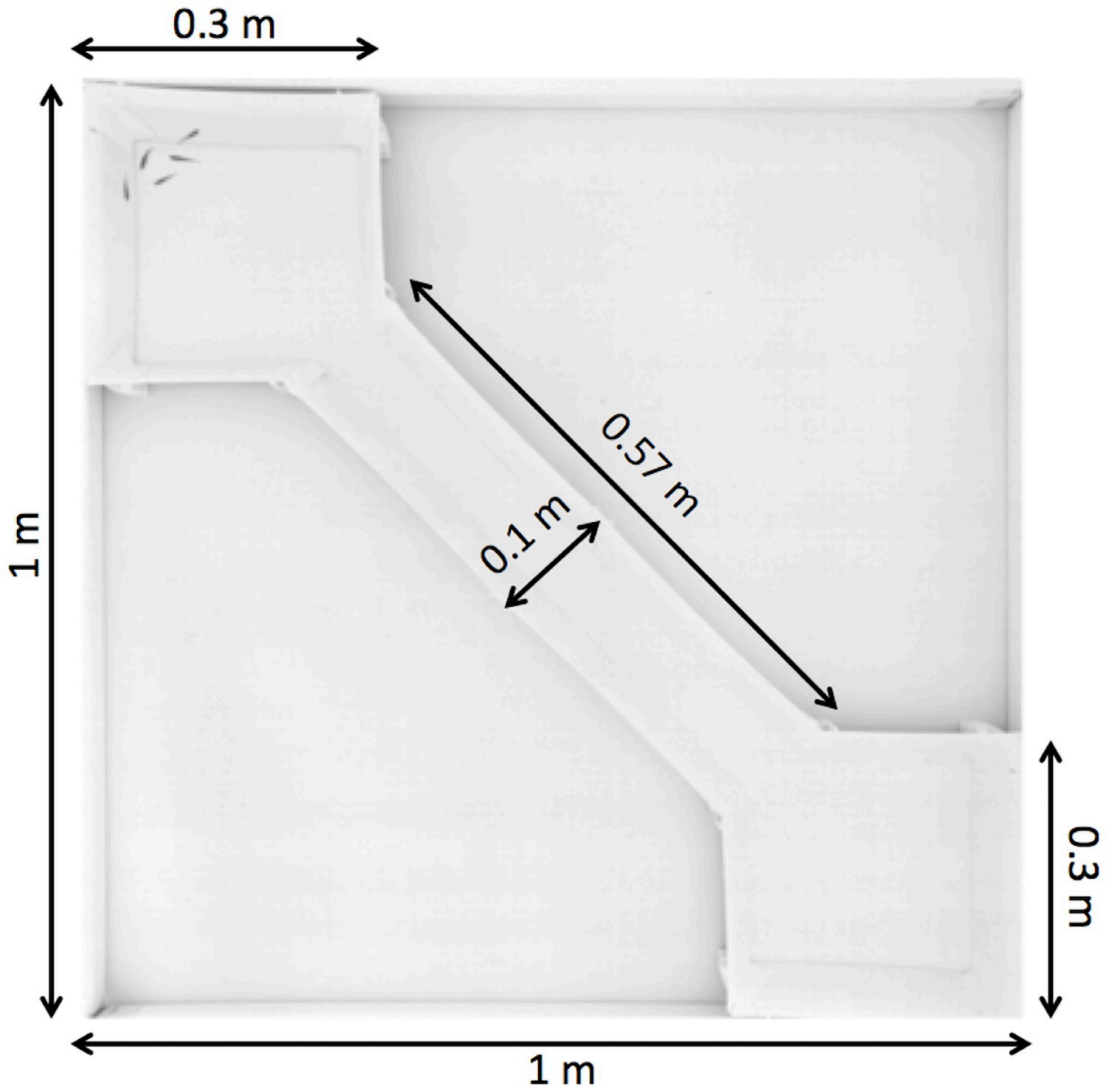
Experimental setup. The arena consists of two square rooms (30 cm x 30 cm) connected by a corridor (57 cm x 10 cm) placed in a 100 cm x 100 cm tank. Twelve groups of 2, 3, 5, 7 and 10 zebrafish were observed swimming freely during trials of 1 hour to study the collective departures of the fish from one room to the other.

We observed shoals of zebrafish swimming in an arena consisting of two square rooms connected by a corridor starting at one corner of each room placed in a 100 cm x 100 cm x 30 cm experimental tank (Fig 1). This experimental set-up allowed us to observe a large number of collective departures for long duration experiments (1 hour) without human intervention. The walls of the arena were made of white opaque PMMA. The water depth was kept at 6 cm during the experiments in order to keep the fish in nearly 2D to facilitate their tracking. One lamp (400W) was placed on the floor at each edge of the tank which is 60 cm above the floor to provide indirect lightning. The whole setup was confined behind white sheets to isolate experiments and homogenise luminosity. A high resolution camera was mounted 1.60m above the water surface to record the experiments at a resolution of 2048 x 2048 pixels and at 15 frames per second.

### Experimental procedure

We observed 12 shoals of two, three, five, seven and ten zebrafish during one hour for a total of 60 experiments. The shoals were formed so that no fish was tested twice the same day and no fish was tested twice for a particular shoal size. Before the trials, the fish were placed with a hand net in a cylindrical arena (20 cm diameter) in one of the two rooms. Following a 5 minutes acclimatisation period, the camera started recording and the fish were released and able to swim in the experimental arena. After one hour, the fish were caught by a hand net to be placed temporarily in a separated tank and replaced in the rearing facilities at the end of the day.

### Tracking

The videos were analyzed offline by the idTracker software [31]. This multi-tracking software extracts specific characteristics of each individual and uses them to identify each fish without tagging throughout the video. This method avoids error propagation and is able to successfully solve crossing, superposition and occlusion problems. However, the tracking system failed to track correctly two experiments with two fish, two experiments with five fish and four experiments with ten fish (some sections of 5 to 10 seconds were missing on the trajectories of one or two fish). Therefore, these four experiments were excluded from our analysis.

In addition, the software provides a confidence probability for the identification of the fish at each time step. We used this identification of the leader (see below).

### Data analysis

For all other experiments, we obtained the coordinates *P(x,y,t)* of all fish as well as a confidence probability for their correct identification by the software at each time step Δt = 1/15s. With these coordinates, we built the trajectories of each fish and computed their position in the arena.

**Fig 2:**
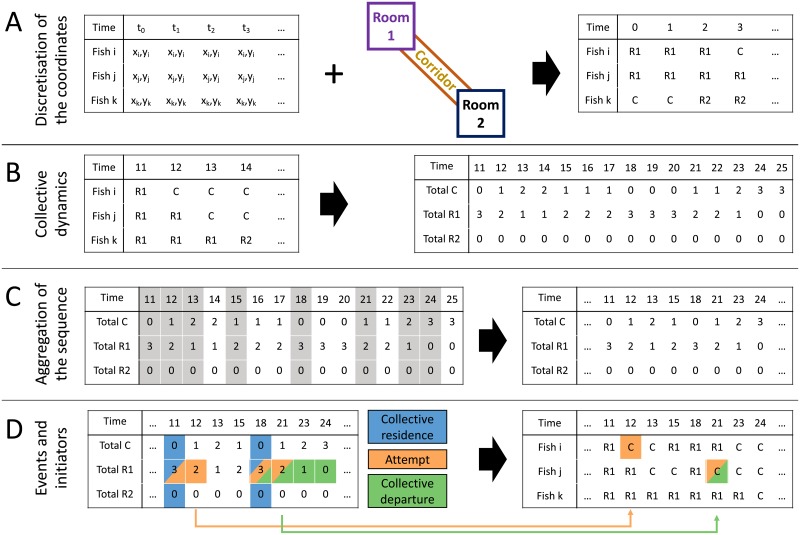
Automated data analysis pipeline of collective departure and leadership. Example for a shoal of three fish. (A) Transformation of the *P(x,y,t)* positions of the fish into symbolic coordinates (C:corridor, R1:room 1, R2:room 2). (B) Computation of the total number of fish in each region. (C) Removal of successive duplicate time steps with no event. (D) Search for particular sequences: three fish in room 1 or 2 (collective residence event), sequences of 3-2 fish in room 1 or 2 (attempt of collective departure) and sequences of 3-2-1-0 fish in room 1 or 2 (collective departure), and identification of the first fish that left the room 1 or 2 for all attempts and successful departures.

To automatically detect collective patterns in the dataset, we developed an automated data analysis pipeline that analyses the trajectories of the fish (Fig 2). First, the algorithm converts the positions *P(x,y,t)* into symbolic coordinates corresponding to the three regions of the experimental arena (Room 1, Corridor and Room 2, Fig 2A). From this derived dataset, the algorithm computes the total number of fish in each zone (Fig 2B). Then, it retains only the time steps at which the movement of a fish occurred (Fig 2C). In this time series, the algorithm searches for particular sequences that correspond to target collective behaviors. As we focused our analysis on the collective departures and the leadership, we targeted three types of event: (i) Collective Residence Events (*CRE*) defined by the entire shoal resting in one of the two rooms, (ii) Attempts of collective departures defined as one fish living one of the two room after a CRE and (iii) Collective Departure Events (*CDE*) defined by the whole shoal leaving one of the two rooms for the corridor after a CRE. For a shoal of *n-fish*, Collective Residence Events are identified by *n-fish* in Room 1 or Room 2. Attempts of collective departure correspond to a successive sequence *n-fish*, *n-fish-1* in Room 1 or Room 2. Finally, collective departures events are identified by a succession of *n-fish*, *n-fish-1*, …, *1*, *0* fish in Room 1 or Room 2. Then, the algorithm identifies the first fish that left the room 1 or 2 for all attempts and collective departures (Fig 2D) and stores their ID and the departure time in two arrays. If more than one fish left the room at the same time step, the algorithm randomly select one of those fish as the leader (this case happened with a frequency of 2% for 2 fish, 2.5% for 3 fish, 14.5% for 5 fish, 6% for 7 fish and 11% for 10 fish). To make sure that we considered only events with a correct identification of the individuals, the algorithm check the confidence probability for the ID the potential leader. If the confidence is higher than 0.75, the candidate fish is definitely considered as the leader of the collective departure or the attempt, otherwise the event is discarded and the algorithm consider the next event of departure (this case happened with a frequency of 3% for 2 fish, 6% for 3 fish, 28% for 5 fish, %15 for 7 fish and 41% for 10 fish). Finally, it computes the total number of attempts and collective departures led by each fish.

Thanks to this analysis, we obtained the total number of collective residence events (*CRE*) and collective departure events (*CDE*) observed for each experiment. To study the effect of the shoal size on these events, we compared the number of events observed for each shoal size but also compared them with measures obtained from simulated non-social shoals in which fish do not pay attention to other shoal members. This null model allowed us to evaluate if any change in the number of collective events detected in our experiments was only due to a scaling effect independently of the social interactions between the animals. To design this non-social shoals, we combined the trajectories of fish originating from different experimental shoals of the same size. Then, we performed the same analysis on this artificial dataset and computed the number of collective residence events (CRE) and collective departure events (CDE).

Then, the distribution of the leadership was quantified by computing the entropy *H(l)* (Eq 1) of the time series of leadership events computed in Fig 2G:

$$\begin{array}{c} H\left(l\right)=-\sum_{i=1}^{k}\frac{L_{i}}{L}\log_{k}\left(\frac{L_{i}}{L}\right) \end{array}$$ (1)

with *k* the number of fish in the shoal, *L_i_* the number of transitions led by the fish *i*, *L* the total number of transitions observed in the experiment and log*_k_* the logarithm to base *k*. In this equation, the ratio *L_i_*/*L* estimates the probability to observe the fish *i* as the leader of a departure. A perfectly uniform distribution of the leadership is associated with the maximal entropy *H(l)* = 1 while a totally despotic organisation is associated with the minimal entropy *H(l)* = 0. Similarly, we studied the temporal organisation of the leaders by computing the conditional entropy *H(l_t_|l_t-1_)*:

$$\begin{array}{c} H\left(l_{t}|l_{t-1}\right)=-\sum_{i=1}^{k}\sum_{j=1}^{k}p\left(l_{t-1}=i,l_{t}=j\right)\log_{k}\left(\frac{p(l_{t-1}=i,l_{t}=j)}{p(l_{t-1}=i)}\right) \end{array}$$ (2)

with *l_t_* the identity of the leader of departure *t* and *l_t-1_* the identity of the leader of departure *t-1*. In this case, the quantity *H(l_t_|l_t-1_)* quantify the ability to predict the identity of the leader *l_t_* of a departure given the identity of the fish *l_t-1_* that led the previous departure. A totally random organisation is associated with the maximal entropy *H(l_t_|l_t-1_)* = 1 while a perfectly predictable organisation results in a minimal entropy *H(l_t_|l_t-1_)* = 0.

Finally, we studied the relationship between the tendency of the fish to initiate a collective departure and their motility. To do so, the instantaneous speed of the fish *v_t_* was computed as the distance between *P(x,y,t-1)* and *P(x,y,t+1)* divided by two time steps.

## Results

### Distribution and temporal organization of the leadership

In all experiments, the fish mainly swam together and regularly transited from one room to the other. In the rooms, the shoal circled a few times before one fish decided to leave the room for the corridor. This fish was either followed by the whole shoal, only a part of the shoal resulting in a temporary split, or not followed at all. In this section, we focused our analysis on the distribution of the leadership and the sequential organisation of the leaders during collective departures from one room to the other. For each CDE, the leader was identified as the first fish of the shoal that left the room. Therefore, we excluded from our analysis the departure of only a subgroup of fish while the rest of the fish remained in the room as well as the departure of a remaining subgroup to join the rest of the shoal.

**Fig 3:**
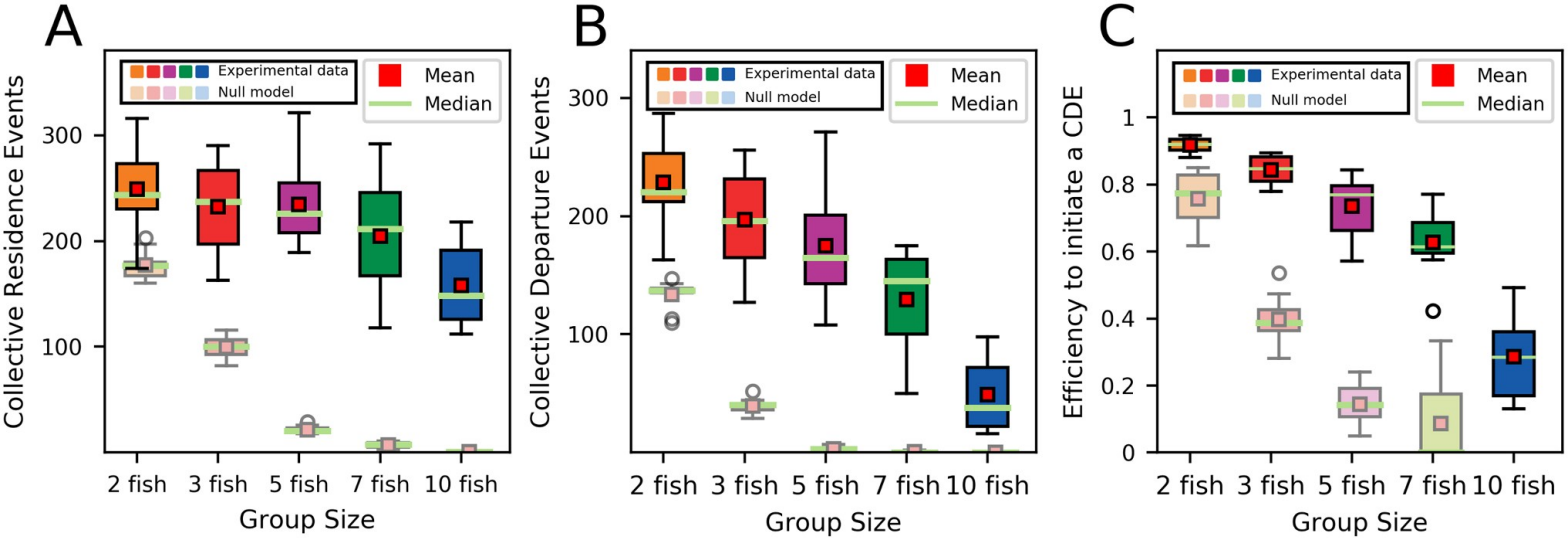
Collective behaviours observed in real shoals (experimental data) and simulated non-social shoals (null-model). (A) Number of collective residence events (CRE) and (B) collective departure events (CDE) for the 11 groups of two, 12 groups of three, 11 groups of five, 12 groups of seven and 10 groups of 10 zebrafish observed during one hour. Collective residence events are defined as the whole group resting in one of the two rooms and collective departures events are defined as the whole group leaving one of the resting sites. (C) Efficiency of the first leaver to trigger a collective departure of all fish computed as the proportion of CRE that were followed by a CDE.

First, we quantified the total number of collective departures events and collective residence events for the different shoal sizes. The number of CRE significantly decreases when the number of fish increases with an average number of 249 ± 38 CRE for two fish to 158 ± 36 CRE for groups of ten fish (Fig 3A, Kruskal-Wallis test, χ^2^ = 15.39, df = 4, p<0.01). Similarly, the number of CDE also decreases but with a stronger difference between the shoals from an average number of 228 ± 35 for two zebrafish to 49 ± 31 CDE for ten zebrafish (Fig 3B, Kruskal-Wallis test, χ^2^ = 33.15, df = 4, p<0.001). Therefore, an average of 92% of the CRE were followed by a collective departure in dyads while only 29% were in groups of ten fish (Fig 3C). This success rate was mainly influenced by the shoal size (p<0.0001) rather than by the number of residence events (p>0.05) that has only a marginally significant effect when coupled with the shoal size (p = 0.03) as shown by the model Succes ∼ shoal size + CRE + shoal size * CRE (Null deviance = 2152.38 with 51 df, Residual deviance = 261.22 with 48 df). Thus, larger shoals were more likely to split during departures while small ones remained cohesive most of the time. However, the shoals remained strongly cohesive compared to simulated non-social shoals. Indeed, the occurrence of these CRE and CDE decreases much more promptly in the null model, with only a few CRE and almost no CDE observed for shoals of five, seven, and ten non-interacting fish. Thus, the smaller decrease in CRE and CDE observed in real shoals compared to non-social agents confirms that the shoals remained strongly social, even with 10 individuals.

**Fig 4:**
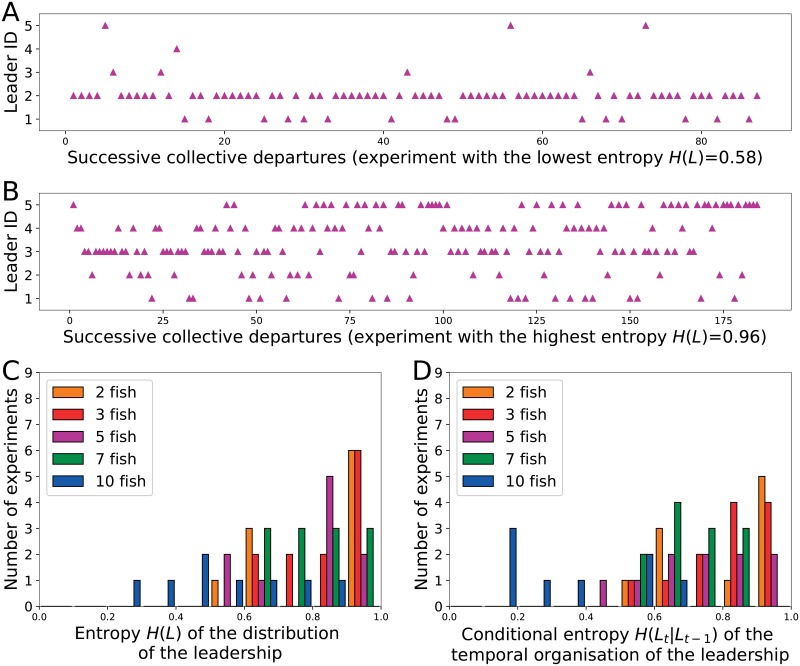
Distribution of the leadership between the shoal members. Sequence of leaders observed for the experiments with the most heterogenous (A) and the most homogeneous (B) distribution of leadership in shoals of five zebrafish. Entropy (C) and conditional entropy (D) computed for all experiments with shoals of 2, 3, 5, 7 and 10 zebrafish.

Thanks to the individual tracking of the fish, we determined the identity of the leaders for all collective departures and computed the sequence of successive leaders’ identities along the experiments. While multiple leaders were observed in each shoal, the leadership was monopolized by only some members of the shoal (e.g. for a shoal of 5 fish Fig 4A) or equally distributed among all individuals (e.g. for a shoal of 5 fish Fig 4B). To better characterise the distribution of the leadership among the fish, we computed the entropy *H(L)* associated with those sequence of leaders observed in each experiment. The distribution of the leadership was significantly influenced by the shoal size (Fig 4C, Kruskal-Wallis test, χ^2^ = 13.77, df = 4, p<0.01). Leadership in small shoals was more likely to be shared among all fish (*H(L)*≈1) while a subgroup of individuals was more involved than others in the leadership for larger shoals (intermediate values of *H(L)*). However, this result also shows that (i) a continuum from homogeneous to heterogeneous sharing of leadership was observed for all shoal sizes, but with different distributions and (ii) no shoal was organized despotically (*H(L)*≈0) with one fish leading all departures, independently of the shoal size. As several initiators were observed in each shoal, we studied the temporal distribution of the leading events to highlight a potential temporal organisation of the leaders over successive departures. To do so, we computed the conditional entropy *H(l_t_|l_t-1_)* normalized for the group size for all sequences of leaders that informs us on the probability to correctly predict the leader of the departure *t* knowing the identity of the leader of the previous departure *t-1*. While no shoal shows a strongly predictable turnover of leaders, the succession of leaders was proportionally less predictable in smaller shoals than in large ones (Kruskal-Wallis test, χ^2^ = 22.94, df = 4, p<0.001). Indeed, the distributions of the conditional entropy were shifted towards lower values as the shoal size increased (Fig 4B). However, the particularly low values observed for 10 fish probably results from the small number of collective departures observed for some shoals (Fig 3D), reducing the potential to observe repeated measures.

**Fig 5:**
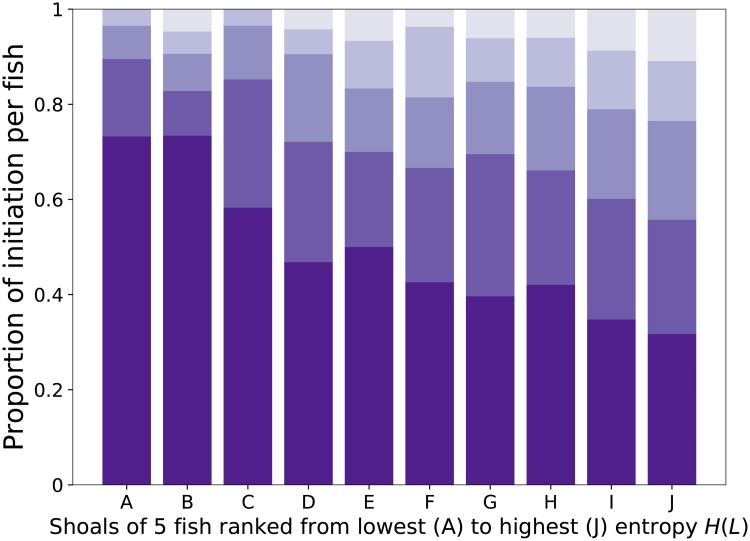
Proportion of departures initiated by each fish in shoals of five zebrafish. The shoals are ranked according to their entropy *H(L)* from the most heterogeneous distribution (left) to the most homogeneous distribution (right). In each shoal, the fish are identified with different colors and ranked from the highest proportion of initiation (bottom) to the lowest proportion of initiation (top). See supplementary material S1 Fig for results with 2, 3, 7 and 10 fish.

These first quantifications at the collective level showed that the leadership during collective departures in zebrafish is shared among the shoal members without a strong temporal organisation. However, they also reveal an inter-shoal variability in addition to a shoal size effect. To highlight this variability, we computed the proportion of collective departures initiated by each fish for each shoal. As expected by the computation of the entropy *H(L)*, we observed a continuum from a homogeneous distribution of the initiation between the shoal members to more heterogeneous ones with some fish having a higher tendency to start a departure than others (Fig 5). Therefore, we studied the individual characteristics of the fish to determine the key factors that influence their propensity to initiate a collective departure.

### Success and profile of the leaders

**Fig 6:**
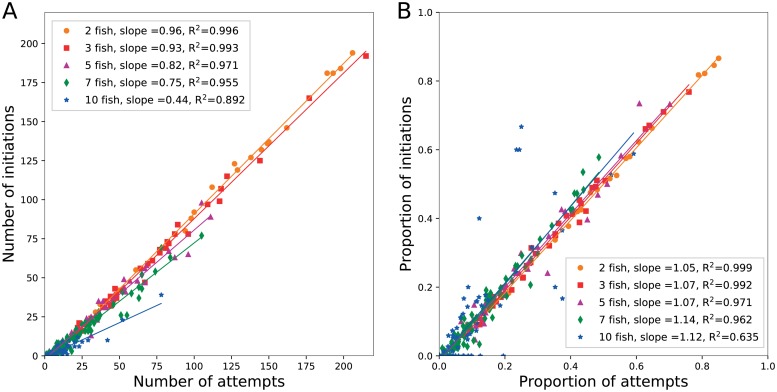
Success rate of the fish. (A) Total number of collective departures initiated as a function of the total number of attempts for each fish. The number of initiations is directly proportional to the number of attempts but the success rate of the initiations decreases for larger group sizes. (B) Proportion of attempts made by the fish in relation to the proportion of departures initiated. For each group size, the success rate is identical for all fish in the shoal.

First, we studied the link between the temperament of the fish and their propensity to initiate collective departures. We determine the boldness of the fish by quantifying the number of times that a fish was the first one to exit a room, independently of its success to be followed by the other shoal members (defined as an *attempt*). For each shoal, we analyzed the potential correlation between the number of attempts and the number of initiations made by each fish. A linear regression showed that the number of initiations is linearly correlated to the number of attempts performed by the fish and that the coefficient of this correlation (i.e. the success rate) depends on the shoal size (Fig 6A). For shoal of two fish, 96% of the attempts made by an individual resulted in a collective departure of the dyad. In accordance with the results observed at the shoal level (Fig 3C), the success rate for each fish decreases when the population increases: 93% for 3 fish, 82% for 5 fish, 74% for 7 fish and 44% for 10 fish. To determine the factors that significantly influence the number of departures initiated by each fish, we designed the GLM *Initiations ∼ shoal size + attempts*. As expected, the shoal size (p<0.0001) as well as the number of attempts made by the fish (p<0.0001) significantly impact the leading success of the fish (Null deviance = 11532.4 with 269 df, Residual deviance = 1880.6 with 267 df). Thus, the attempts made by the fish were significantly more successful in small shoals than in larger ones.

Then, we analysed the success of the fish by comparing their intra-shoal proportion of initiated departures with the intra-shoal proportion of attempts that they made. A linear relationship would imply that all fish shared the same success rate, independently of the number of attempts that they made while a wider dispersion or a non-linear relationship would highlight that some fish have a higher success rate than others. As shown in Fig 6B, the two proportions are linearly correlated for all shoal sizes with a slope ≈ 1, revealing an equal success rate for all shoal members. This conclusion is supported by the model *Proportion of initiations ∼ shoal size + proportion of attempts* identifying only the intra-shoal proportion of attempts (p<0.0001) made by a fish as a significant predictor of the proportion of collective departures that it initiated with no influence of the shoal size (p = 0.648) on its success (Null deviance = 7269.78 with 269 df, Residual deviance = 521.27 with 267 df). Thus, the larger number of departures initiated by some fish is not related to a higher influence on other group members or a better success rate but on a higher tendency to exit the resting sites.

**Fig 7:**
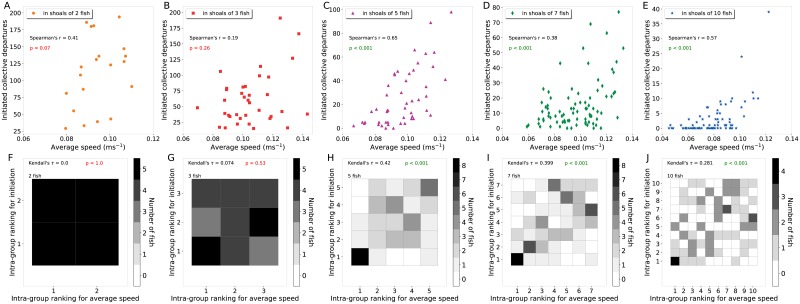
Motility of the fish and leadership. (A-E) Proportion of attempt made by each fish according to its average speed. For small group sizes (2 or 3 fish), the absolute value of the mean linear speed is not a good predictor of the number of attempts performed by a fish (Sperman’s correlation) but for larger group size (5, 7 and 10 fish), the linear speed of a fish is statistically correlated with the number of attempts. (F-J) Distribution of the fish according to their intra-group ranking of the number of collective departures that they initiated and their intra-group ranking for the average speed. By taking into account their ranking inside the group, the relationship is statistically significant as soon as the groups is formed by at least five fish.

Finally, we looked at a potential link between the motion characteristics of the fish and the number of collective departures that they have initiated. To do so, we measured the average linear speed of all individuals and also compared their intra-group ranking for the number of initiations with their intra-group ranking for the linear speed using the Kendall’s *τ* coefficient. For small shoals (two and three fish), we found no correlation between the average speed of the fish and their tendency to lead a departure (Fig 7A and 7B for global comparisons and & F-G for intra-group comparisons). On the contrary, there was a significant positive correlation between the average speed of the fish and the number of initiations that they performed for shoals of 5, 7 and 10 fish (Fig 7C–7E). Indeed, for the majority of the shoals (8 out of 10 for 5 fish; 8 out of 12 for 7 fish and 4 out of 8 for 10 fish) the individual that initiated the largest number of collective departures was also the one with the highest average speed (Fig 7H–7J). Thus, the initiation of collective movements is related to the motility of the fish but only for large shoals.

## Discussion

The coordinated movements of fish shoals are often reported as a collective process in which each fish can potentially lead a departure. Here, we confirmed that the initiation of collective departures is a distributed process among the shoal members. However, our results also showed that the sharing of the leadership across the different groups was a continuum from a homogeneously distributed leadership to strongly asymmetrical distributions. A similar diversity was observed in groups of four zebrafish during foraging [31]: in two groups out of four, the order of arrival was consistent over successive trials while the fish in the two other groups showed a random arrival order. In our experiments, dyads showed the most egalitarian situations but also the strongest monopolization of leadership with one fish performing up to 85% of the initiations of its shoal. A similar result was observed in trios with some shoals sharing equally the leadership between all members and other groups with a disproportionate number of initiations led by the same fish (up to 75% for one group). As the shoal size increases, almost all shoals showed a heterogeneous distribution of the leadership between the fish even if we did not observe a clear monopolization of the initiations of collective departures in these shoals. A similar effect of group size on leader-followers interaction was evidenced in minnows [36]. In this latter study, 6 out of 9 dyads displayed a clear leader-follower relation, 2 showed an equally shared leadership and 1 was formed by fish that did not interact with each other. The author concluded that one fish leads the other in groups of two but that this behaviour is not observed for larger groups.

Stronger asymmetries are more likely to be observed in small group sizes but an unbalanced distribution is almost always present in groups of a dozen of individuals. Such outcome can be the result of sampling of a continuous distribution for an individual characteristic that influences the probability to lead the group. Indeed, as we add more individuals, there is a higher probability that at least two of them significantly differ from each other, leading to an unshared decision-making process but by the same time, the average difference between individuals tends to stabilize to a limit value. On the contrary, as only two fish are forming a dyad, there is a probability that these fish are either almost identical, resulting in a homogeneous leadership, or on the contrary strongly different, leading to a heterogeneous leadership, with a continuum of possibilities between these extrema. For example, the boldest individual of a dyad tend to take the lead more often than the shier one in pairs of sticklebacks, this tendency being amplified for greater difference in boldness between the fish [28].

Incidentally, one should be cautious when subdividing the tested population into binary behavioural classes and then performing experiments with pairs of opposed individuals. Indeed, such classification may lead to a misrepresentation of the social organisation observed in free-living groups by forming only asymmetrical pairs. This effect was already mentioned by [37] that concluded that a significant relation between boldness and leadership in golden shiners only when they classified the fish into the binary *leaders* or *non-leaders* classes. Thus, social species in which the initiation of specific behaviours is related to individual characteristics rather than to a particular hierarchical position, are more likely to display a whole range of social structures from despotic to egalitarian groups without any behavioural changes but only due to sampling effects.

Thanks to the individual tracking of repeated departures, we also highlighted that the heterogeneous distribution of leadership was not the result of a higher success rate of some individuals that could have a higher tendency to be followed. On the contrary, the number of successful departures initiated by the fish was linearly correlated to their number of attempts. In addition, we showed that the initiation process was not temporarily organised as the identity of the fish that led a departure does not provide information on the identity of the fish leading the next one. A similar result was also observed in other fish species like Damselfish in which collective departures from one spot to another was mainly led by fish that performed a higher number of attempts [38]. In sticklebacks, shy individuals tend to be more responsive to the departure of another shy fish while bold individuals are less sensible to the personality of the initiator [39]. Nevertheless, bold individuals are more likely to be observed as leader simply because they try to initiate more collective movements.

While the linear relation between the number of attempts and initiations was observed for all group sizes in our experiments, the success rate of the attempts drops from ≈90% in dyads to only ≈20% in groups of ten fish. The majority of attempts led to a temporary fission of the shoal into subgroups for this larger group size. In our experimental setup, the subgroups always reassembled after a short period of time. However, in natural conditions where the fish are not restrained to a small environment, those splitting events could lead to a permanent fission of the group. Indeed, zebrafish form shoals of a few to dozens of individuals in their habitat [40]. The size of the shoals observed in nature can be driven by a trade-off between the advantages (e.g. detection of predators and potential food sources) and disadvantages (e.g. larger groups are more easily spotted by predators, increased inter-individual competition for food) of being in groups. According to our results, the intermediate shoal sizes observed in zebrafish could be maintained by a strong cohesion in small groups but a loose organisation of larger ones making them more prone to splitting.

Our results also highlighted that the motility of the fish was a predictor of its tendency to initiate collective departures for larger groups. Indeed, the intra-group ranking of a fish for the average speed was correlated to its intra-group ranking for the number of led departures for shoals of five, seven and ten fish. In those shoals, the leaders of collective movements do not seem to occupy a particular hierarchical status in the group but are generally the most mobile individuals. A similar result was predicted by a theoretical analysis on the emergence of leadership in simulated zebrafish [41]. This study showed that an informed individual moving in a specific direction is more likely to be followed by a group of naive individuals when it moves just faster than the naive group. In Damselfish *Dascyllus aruanus*, the initiator of a collective movement also displays a higher level of activity than their group members before the departure [38]. A similar result was observed during pigeon flocking: birds with the highest ground speed tend to lead the flock more often than others [42]. A favored direction, a higher level of activity or a higher average speed can lead an individual to occupy the front position of the group more often than other individuals. As the direction of the group is mainly decided by the front individuals (at least in shoals of fish [18]), these inter-individual behavioural differences lead to a heterogeneously distributed leadership in the shoal.

The present study demonstrated that any fish could potentially lead the collective movements of its shoal, with the same success rate for all shoal members. Such democratic organisation may enhance the transfer of information between shoal members. Indeed, the fish did not differ in their needs or in their knowledge about the environment in the experiences described in this paper. However, in a natural context, some fish may differ in the level of information that they have about environmental opportunities or threats. In this context, it seems more adaptive to adopt a collective decision-making process that allows any group member to initiate a collective decision or movement. Nevertheless, we also showed that even if the process allows any individual to act as a leader, some individuals do so more often than others. This variation in the tendency to initiate a collective movement or to follow other shoal members could tend to stabilize over time and lead to specialized roles. Indeed, if initiation is linked to individual personality traits such as boldness, individuals could repeatedly experience the same role in decision making process (i.e. initiator or follower). This division of labour in decision-making may be advantageous as some member may become more incline to propose new directions of movement that can be evaluated and then approved or rejected by other group members.

This may be a primitive division of labour in collective information processing and decision-making. Initiators may be bolder individuals that react more promptly and less carefully to their environment while followers may be more cautious in their decision. This balance between bold initiators and shy followers may prove to be advantageous for group living species, as initiators will bring possibilities and followers may help to select the best options proposed as well as maintain social cohesion. Indeed, it has been shown that the presence of naive followers in fish may maintain the social cohesion of their shoal when some shoal members have conflicting information about the direction to adopt [24]. This process may be particularly adaptive for collective decision-making in groups that share the same interests as each individual is able to express an opinion but also rely on the approval of the majority of the group members.

## Supporting information

S1 Fig Proportion of departures initiated by each fish for shoals of 2, 3, 7 and 10 zebrafish. The shoals are ranked according to their entropy *H(L)* from the most heterogeneous distribution (left) to the most homogeneous distribution (right). In each shoal, the fish are identified with different colors and ranked from the highest proportion of initiation (bottom) to the lowest proportion of initiation (top).
(EPS)
